# Microscopic elucidation of abundant endophytic bacteria colonizing the cell wall–plasma membrane peri-space in the shoot-tip tissue of banana

**DOI:** 10.1093/aobpla/plt011

**Published:** 2013-02-22

**Authors:** Pious Thomas, Krishna M. Reddy

**Affiliations:** 1 Division of Biotechnology, Indian Institute of Horticultural Research, Hessarghatta Lake, Bangalore 560089, India; 2 Division of Plant Pathology, Indian Institute of Horticultural Research, Hessarghatta Lake, Bangalore 560089, India

**Keywords:** Apoplast, confocal laser scanning microscopy, endophytes, fluorescence microscopy, Live-Dead bacterial staining, micropropagation, *Musa* sp., plant cell biology

## Abstract

This study was aimed at generating microscopic evidence of intra-tissue colonization in banana in support of the previous findings on widespread association of endophytic bacteria with the shoot tips of field-grown plants and micropropagated cultures, and to understand the extent of tissue colonization. Leaf-sheath tissue sections (∼50–100 µm) from aseptically gathered shoot tips of cv. Grand Naine were treated with Live/Dead bacterial viability kit components SYTO 9 (S9) and propidium iodide (PI) followed by epifluorescence or confocal laser scanning microscopy (CLSM). The S9, which targets live bacteria, showed abundant green-fluorescing particles along the host cell periphery in CLSM, apparently in between the plasma membrane and the cell wall. These included non-motile and occasional actively motile single bacterial cells seen in different *x*–*y* planes and *z*-stacks over several cell layers, with the fluorescence signal similar to that of pure cultures of banana endophytes. Propidium iodide, which stains dead bacteria, did not detect any, but post-ethanol treatment, both PI and 4′,6-diamidino-2-phenylindole detected abundant bacteria. Propidium iodide showed clear nuclear staining, as did S9 to some extent, and the fluorophores appeared to detect bacteria at the exclusion of DNA-containing plant organelles as gathered from bright-field and phase-contrast microscopy. The S9–PI staining did not work satisfactorily with formalin- or paraformaldehyde-fixed tissue. The extensive bacterial colonization in fresh tissue was further confirmed with the suckers of different cultivars, and was supported by transmission electron microscopy. This study thus provides clear microscopic evidence of the extensive endophytic bacterial inhabitation in the confined cell wall–plasma membrane peri-space in shoot tissue of banana with the organisms sharing an integral association with the host. The abundant tissue colonization suggests a possible involvement of endophytes in the biology of the host besides recognizing cell wall–plasma membrane peri-space as a major niche for plant-associated bacteria.

## Abstract (precis)

Plants are known to harbor endophytic bacteria, the organisms residing internally without imparting any apparent adverse effects on the host. Endophytes are generally known to be present in few numbers colonizing the intercellular spaces, primarily in roots. This study adopting SYTO 9 staining and live confocal imaging of fresh tissue sections from the shoot-tip region of banana, supported by transmission electron microscopy, brings out, possibly for the first time, extensive bacterial colonization in the confined cell wall – plasma membrane peri-space. The integral host-association and their abundance suggest a prominent role of endophytes in the biology of the host.

## Introduction

The plant microbiome includes pathogenic, symbiotic, epiphytic and endophytic associations (Parniske 2000; Rosenblueth and Martínez-Romero 2006). Endophytes are known to colonize plants internally, often in the intercellular region, without imparting any adverse effects (Hallmann *et al.* 1997; Bacon *et al.* 2002; Compant *et al.* 2010; Reinhold-Hurek and Hurek 2011). Bacterial endophytes have been documented in diverse plant species and organs (Hallmann *et al.* 1997; Thomas *et al.* 2007*a*; Mano and Morisaki 2008; Reinhold-Hurek and Hurek 2011; Kaewkla and Franco 2013). Endophytic bacteria are becoming increasingly recognized in crop production on account of their potential utility as agents in plant growth promotion, stress alleviation and phytoremediation (Thomas *et al.* 2007*a*; Backman and Sikora 2008; Doty 2008; Hardoim *et al.* 2008; Ryan *et al.* 2008).

Banana (*Musa* sp.) forms a major fruit and food crop worldwide (Singh *et al.* 2011). The true stem in this herbaceous plant is the underground corm, while leaf sheaths constitute the pseudostem. The deep-seated shoot tip, protected from the exterior by numerous leaf bases, forms the starting material for micropropagation in banana, a practice now commonly adopted for the rapid clonal multiplication of elite types (Thomas *et al.* 2008*a*; Singh *et al.* 2011). Studies employing tissue-cultured and field-grown bananas have indicated the widespread association of bacterial endophytes predominantly in a non-culturable form, and also in a culturable but non-obvious or covert form in the micropropagated stocks (Thomas *et al.* 2008*a*; Thomas and Soly 2009). The culturable organisms isolated from the popular cv. Grand Naine consisted of nearly 50 species within the classes of α-, β- and γ-proteobacteria, firmicutes and non-filamentous actinobacteria (Thomas *et al.* 2008*b*; Thomas and Soly 2009). Microscopic observations on the tissue homogenate of banana had indicated the presence of bacterial cells in substantial numbers, which, supported by molecular data, suggested that the organisms detected as colony-forming units on nutrient media constituted only a minor fraction of the total population and that the majority of cells were refractory to cultivation (Thomas *et al.* 2008*a*, *b*; Thomas and Soly 2009).

The ultimate proof of tissue colonization by endophytes comes from microscopic documentation (Gyaneshwar *et al.* 2001; Compant *et al.* 2008, 2011; Thomas 2011). Earlier efforts to localize the organisms inside the banana shoot tissue employing conventional tissue fixation, microtomy and staining were limited by the high background and the inability to differentiate the bacterial cells from the tissue constituents or cellular inclusions (Thomas *et al.* 2008*a*; Thomas and Soly 2009). Currently adopted approaches to visualize the native endophytic bacteria in plant tissue include transmission electron microscopy (TEM) (Gyaneshwar *et al.* 2001), fluorescent *in situ* hybridization (FISH) (Compant *et al.* 2011) and triphenyl tetrazolium chloride vital staining (Bacon *et al.* 2002; Thomas 2011), while tagging with labels such as green fluorescent protein (GFP) facilitates the monitoring of externally applied organisms (Compant *et al.* 2008; Prieto *et al.* 2011).

Bacterial monitoring with the Live/Dead bacterial viability kit (Molecular Probes^®^) comprising the fluorophores SYTO 9 (S9) and propidium iodide (PI) is now employed in different spheres of microbiology (Anonymous 2004; Berney *et al.* 2007; Gião *et al.* 2009). While live bacteria with intact cell membranes are stained fluorescent green by S9, those with damaged membranes are stained red by PI (Anonymous 2004). To our knowledge, this approach has not been explored much in studies of plant–microbe association except for some isolated reports (Böhm *et al.* 2007; Lucero *et al.* 2011). In this study, we demonstrate the application of the Live/Dead bacterial viability kit with confocal imaging on fresh tissue sections as a simple and efficient tool for documenting native endophytic bacteria, and also elucidate the extensive bacterial colonization in the peri-space between the cell wall and the plasma membrane in the growing shoot-tip region of banana.

## Methods

### Banana genotypes and tissue preparation

Field-derived 2- to 4-month-old suckers of cv. Grand Naine formed the main experimental sample. The shoot tips comprising the apical 2–3 cm of pseudostem sheaths and the anchoring 1 cm corm tissue were excised aseptically after extensive surface sterilization as per Thomas *et al.* (2008*a*). The tissue was fixed in 4 % formalin–phosphate-buffered saline (PBS) or 2 % paraformaldehyde–PBS overnight at 4 °C. Thin tissue sections prepared from the innermost sheaths of fixed tissue as well as fresh tissue from 10 suckers each were employed in bright-field, epifluorescence and confocal microscopy. Five suckers each from the cvs. Robusta, Dwarf Cavendish and Ney Poovan were used for validation. In addition, micropropagated stock cultures of cv. Grand Naine were also employed. These included five from the stocks showing non-obvious or covert bacterial association as brought out through tissue indexing (Thomas *et al.* 2008*a*), and five from stocks not showing culturable bacteria but non-culturable organisms as per the microscopic observation of the tissue squeeze under phase contrast as described elsewhere (Thomas *et al.* 2008*a*; Thomas and Soly 2009).

### Bright-field and phase-contrast microscopy

A Leica DM2000 optical microscope along with a DFC-295 digital live camera and Leica Application Suite (LAS) software version 2.0 (Leica Microsystems CMS GmbH, Wetzlar, Germany) was employed for bright-field and phase-contrast microscopy. Thin, free-hand-cut tissue sections (∼50–100 µm) prepared employing a fine razor blade, as well as the tissue homogenate, were examined after mounting on acetone-washed and autoclaved microscope slides under oil immersion (×1000). The images were captured with LAS software and further processed with Adobe Photoshop 7.0 (Adobe Systems Inc., San Jose, CA, USA) software.

### Epifluorescence microscopy

4′,6-Diamidino-2-phenylindole (DAPI; Sigma Chemical Co., St Louis, MO, USA) 10 µg mL^−1^ stock and the Live/Dead bacterial staining kit L13152 (Molecular Probes^®^, Life Technologies, New York, NY, USA) comprising S9 and PI prepared in molecular biology grade sterile water were used as per the manufacturer's instructions (Anonymous 2004). Thin sections (∼50–100 µm) were prepared aseptically from fixed as well as fresh tissue. These were covered with the fluorophores singly or in combination as per the manufacturer's instructions (6 μM S9 and 30 μM PI at 1×) and examined under the epifluorescence microscope after 10–20 min. A Leica DMLB2 microscope with GFP and I3 filter cubes for blue excitation, and N3 for green excitation, and with a DFC320 digital live camera (Leica Microsystems CMS GmbH, Wetzlar, Germany) under ×63 oil or ×100 water immersion objectives was employed. The images were captured with LAS software version 2.0 and further processed with Adobe Photoshop 7.0. The preparation of S9 stain or the mounting of tissue in phosphate buffer (0.5 M; pH 7.4), PBS or saline versus plain distilled water was also assessed for the signal levels.

### Confocal laser scanning microscopy

Thin sections prepared from fresh tissue as above were examined 10–20 min after the application of DAPI, S9 or PI fluorophores singly, or in combinations of two fluorophores, using an LSM 5 LIVE confocal laser scanning microscope (CLSM) equipped with a 488 nm laser and supported by LSM software (Carl Zeiss Inc., Jena GmbH, Germany). SYTO 9- and PI-stained samples were excited at 488 nm, and the DAPI-stained samples at 405 nm. Confocal stacks/data were analysed using the Zeiss LSM Image Browser version 4.0 program (Carl Zeiss Inc.). Image J was used to generate avi files from time-lapses and *z*-stacks. Video files were also assembled with the images from different time-lapses or *z*-stacks using Microsoft Windows Movie Maker software. Two-dimensional images were processed with LSM image browser, MS-Power Point and Adobe Photoshop 7.0.

### SYTO 9 staining of pure cultures of bacteria

Pure cultures of bacteria isolated from banana as endophytes (Thomas *et al.* 2008*b*, Thomas and Soly 2009) were used in CLSM after staining with S9 to assess their similarity to the bacteria detected in fresh tissue sections. These included *Brachybacterium*, *Micrococcus*, *Kocuria* and *Staphylococcus* spp. representing the cocci group, *Brevibacterium*, *Microbacterium* and *Tetrasphera* spp. with fine rods, *Enterobacter cloacae* with medium long rods, and *Bacillus subtilis* with longer rods and spores.

### Transmission electron microscopy

Tissue was fixed in glutaraldehyde (5 %)–osmium tetroxide (1 %), dehydrated with an acetone series and propylene oxide, embedded in epoxy SPURR medium and sections of ∼1 µm prepared (Ultra cut Model S; Richards, UK). After a further dissection to 70 nm, sections were stained with uranyl acetate (2 %) and lead citrate (2 %) for 5 min each and scanned with a JEOL 100S (Japanese Electronic Opticals Ltd, Japan) TEM or a Tecnai™ G2 Spirit BioTWIN TEM fitted with a Gatan Orius 1000 camera.

## Results

### Bright-field and phase-contrast microscopy

Under bright field/phase contrast, the host cells showed intact organelles like plastids and mitochondria with no detectable intercellular spaces or large vacuoles [**seeSupporting Information–Fig. S1**]. Tissue sap/squeeze showed abundant bacterial cells amidst plastids and mitochondria under bright field and more obviously under phase contrast, characterized by their active motility or the typical wriggling movement as documented earlier (Thomas *et al.* 2008*a*; Thomas and Soly 2009).

### Epifluorescence microscopy

Staining of tissue sections from the inner sheaths of formalin- or paraformaldehyde-fixed shoot-tip tissue with DAPI showed the nuclei clearly under an epifluorescence microscope but no bacterial detection. With S9 and PI, the formalin-fixed tissue showed high auto-fluorescence with no obvious detection of bacteria.

**Figure 1.**
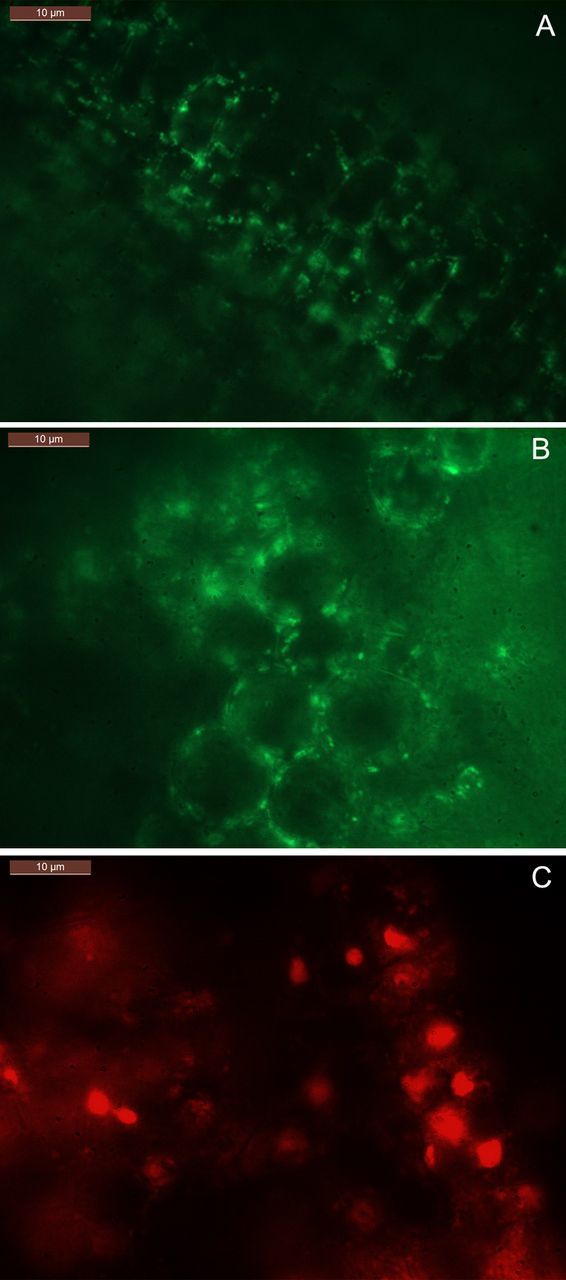
Epifluorescence microscopy. Fresh tissue sections from the shoot-tip explants of banana cv. Grand Naine stained with S9 displaying abundant small green-fluorescing bacterial cells internally along the cell periphery (A, B) and nuclear staining by PI (C) under the ×63 objective of an epifluorescence microscope (horizontal bar = 10 µm).

Based on the observation that pure cultures of bacteria prepared in distilled water yielded a clear fluorescence signal with S9 compared with formalin-fixed cells, tissue sections from fresh non-fixed inner leaf sheaths were considered. Epifluorescence microscopy after S9 treatment of such tissue sections indicated small green-fluorescing bacterial cells in abundant numbers mainly along the cell periphery but internal to the cell wall, while PI showed red-fluorescing nuclei (Fig. 1A–C). Other DNA-containing structures including nuclei, plastids and mitochondria were not easily detected with S9. This was evident from the staining of nuclei with DAPI or PI, and the revelation of organelles under phase contrast.

While S9 staining worked fine with fresh tissue sections mounted in molecular biology grade water facilitating the detection of abundant bacteria along the cell periphery, tissue fixed in formalin or paraformaldehyde displayed a faint or no fluorescence signal **[see Supporting Information–Fig. S2]****.** Furthermore, the mounting of tissue or preparing the dye in PBS or PO_4_ buffer also interfered with S9 staining, with no detection of bacteria at all while employing 0.5 M PO_4_ buffer and some diffuse staining in PBS. The presence of salts interfered with S9 labelling, causing rapid decay of signal or a high background fluorescence. No auto-fluorescence was observed with untreated fresh tissue under a GFP filter.

### Confocal laser scanning microscopy of Grand Naine tissue

**Figure 2.**
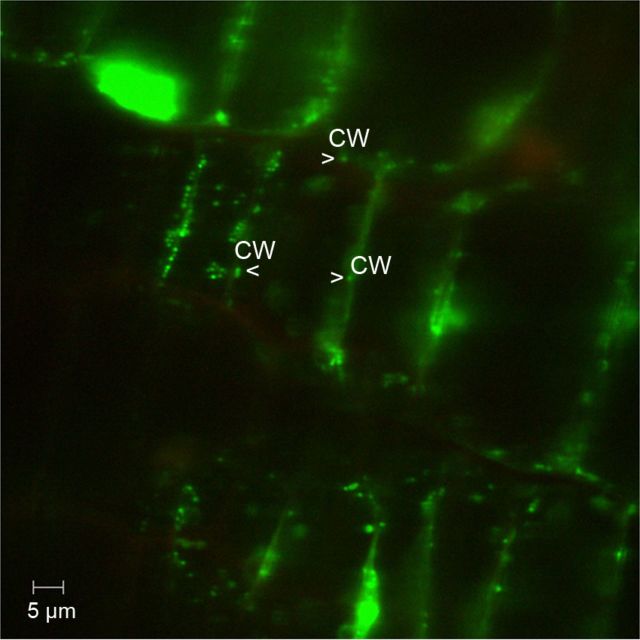
Confocal imaging after S9 staining of tissue sections of banana. Confocal imaging of S9 applied to fresh tissue segment of cv. Grand Naine displaying abundant green-fluorescing bacteria (indicated by arrowheads) just inside the cell wall (cw) along the cell periphery in the *x*–*y* plane.

Confocal examination of fresh tissue sections following S9 treatment supported the observations from epifluorescence microscopy detecting abundant bacteria along the host cell periphery. In addition, CLSM showed that the fluorescing particles were confined in the extra-cytoplasmic area of the cell, i.e. in between the plasma membrane and cell wall (Fig. 2). Time-lapse examination revealed that the fluorescing bacterial cells were either single non-motile units or occasionally moving particles confined to the peri-cytoplasmic region in the *x*–*y* plane, as demonstrated through videography **[see Supporting Information–Videos 1–3]**. Some bacterial cells *prima facie* appeared to be inside the cell matrix, but were possibly the ones that were released during tissue disruption or those that were captured near the cell exterior in a horizontal plane, as the video-recordings indicated confined movement along the cell periphery. The possibility of intra-cellular bacteria, however, could not be ruled out. Conversion of CLSM time-lapse data of ≥30 s to avi files, employing Image J, gave video files five times faster than the actual one. Therefore, the motility rate observed with the video files is different from the real-time situation.

**Figure 3.**
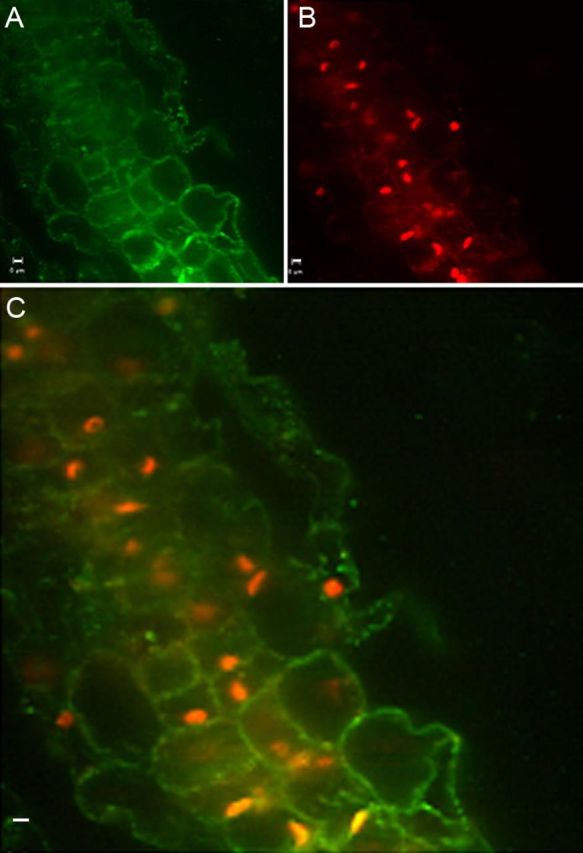
Combined use of S9 and PI. Use of S9 together with PI on free-hand-cut tissue section from fresh leaf sheaths of banana cv. Grand Naine, showing bacteria along the cell peri-space with S9 (A); nuclear staining by PI (B); the superimposed image (C) shows both stained bacteria and nuclei (horizontal bar = 5 µm).

Confocal *z*-stacking over several cell layers to a depth of 25–50 µm revealed extensive tissue colonization along the cell boundary **[see Supporting Information –Fig. S3; Videos 4 and 5]**, while PI and DAPI treatments did not detect them. Some nuclear staining was detected with S9 under confocal imaging after extended treatment but not of the DNA-containing organelles, namely plastids and mitochondria, which were otherwise obvious under phase contrast. The combined use of S9 and PI showed bacterial staining with the former and nuclear staining by the latter (Fig. 3). Control tissue sections without the fluorophores did not show any detectable signal under confocal imaging. As with epifluorescence microscopy, preparation of the stain or mounting of tissue in PO_4_ buffer, PBS or saline resulted in nil or reduced signal levels with S9 compared with its use in distilled water.

### Confocal laser scanning microscopy of pure bacterial cultures

Confocal examination of pure cultures of different bacteria that were isolated as endophytes from banana confirmed their similarity to the extra-cytoplasmic bacteria in terms of gross green fluorescence after S9 staining under the same magnification **[see Supporting Information–Fig. S4]**. Bacterial cells with different sizes or shapes often did not show such a clear size- or shape-based discrimination at the magnification levels employed. For instance, *Tetrasphaera* and *Brevibacterium* with small rods showed an intense signal, while *Enterobacter* with larger rods, and cocci of *Kocuria* sp., yielded lower signals. The large green-stained bodies in the tissue images after S9 treatment appeared to result from several bacterial cells remaining in close proximity outside the focal plane.

### Confocal laser scanning microscopy of ethanol-treated tissue and other genotypes

**Figure 4.**
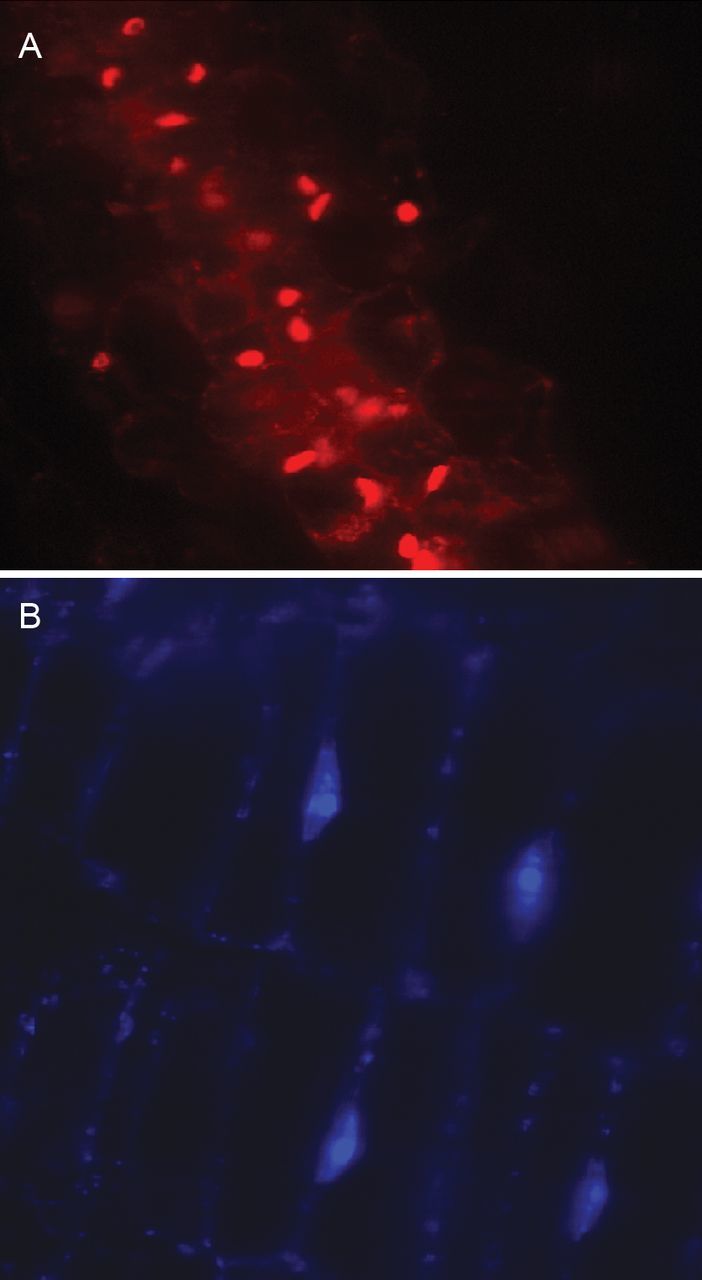
Propidium iodide and DAPI staining after ethanol treatment of tissue. Thin free-hand-cut tissue segments of fresh leaf sheaths treated with 90 % ethanol followed by staining with DAPI 10 µg mL^−1^ stock (A), or PI from the Live/Dead bacterial viability kit (B) showing nuclei and bacteria along the cell boundary.

Tissue segments treated with 90 % ethanol, which induces pores in bacterial cell membranes, displayed staining of extra-cytoplasmic bacteria with PI and DAPI besides the nuclei (Fig. 4). No staining of plastids or mitochondria was observed with S9, PI or DAPI even after ethanol treatment.

**Figure 5.**
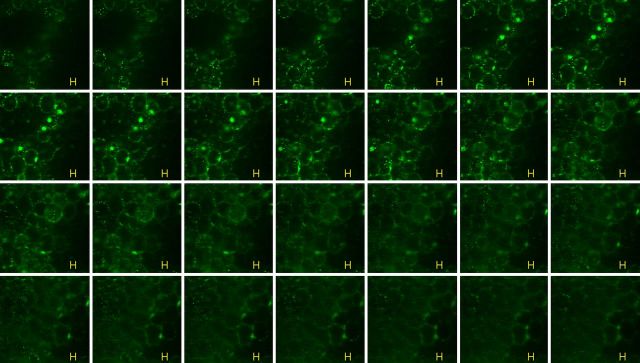
Confocal *z*-stacks of cv. Robusta tissue sections after S9 staining. Thin free-hand-cut tissue segments of fresh leaf sheaths stained with S9 showing abundant peri-space bacteria in different cell layers over 54 µm depth at 2-µm intervals (serial stacks from the left to right; horizontal bar = 10 µm).

The above-described observations on the extensive peri-cytoplasmic colonization by the endophytic bacteria were proven true with Grand Naine suckers collected from different locations and with the other cultivars, Robusta (Fig. 5) **[see Supporting Information–Video 6]**, Dwarf Cavendish and Ney Poovan **[see Supporting Information–Fig. S5]**. Micropropagated stocks also showed abundant bacteria in the intra-cell wall region of shoot tissue irrespective of being index positive for culturable bacteria, or harbouring non-culturable bacteria, being index negative on enriched bacteriological media (data not shown).

### Transmission electron microscopy

**Figure 6.**
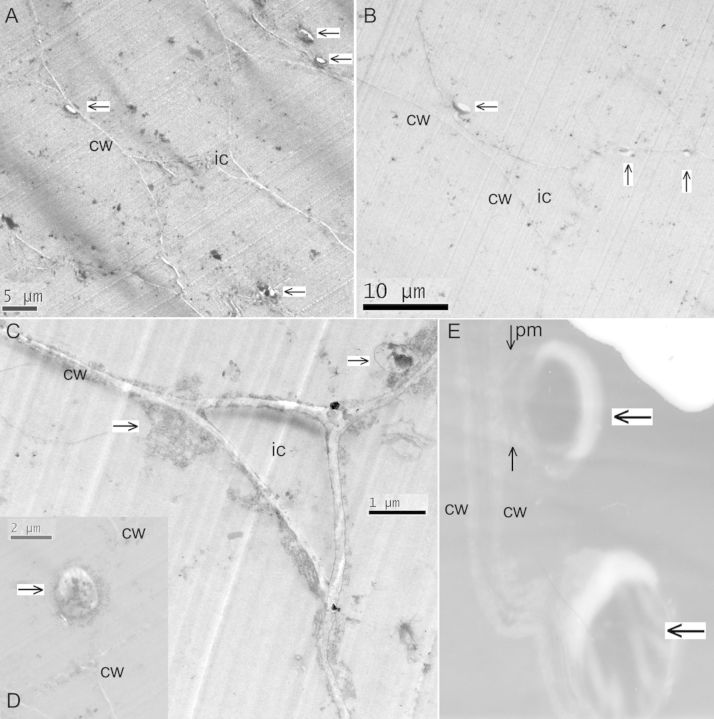
Transmission electron microscopy on tissue sections of cv. Grand Naine specifically stained for bacteria showing bacterial cells (indicated by an arrow) just inside the cell wall (cw) adjoining the plasma membrane captured at ×1400 (A), ×1200 (B), ×13 000 (C) or ×4800 (D) with a Tecnai™ G2 Spirit BioTWIN TEM, or at ×14 000 (E) using a JEOL 100S TEM with a further ×2 magnification in Adobe Photoshop 7.0. Intercellular space (ic) is obvious in (A), (B) and (C), and the plasma membrane (pm) envelope originating from the cell wall is indicated by the thin black arrow marked in ‘E’.

Transmission electron microscopy observations as per the staining procedure adopted herein clearly showed that the bacterial cells were internal to the cell wall and not exterior to it (Fig. 6). At higher magnifications, bacterial cells could be seen close to the cell wall, held in place by the stretched plasma membrane (Fig. 6E).

## Discussion

This study using the Live/Dead bacterial viability kit uncovers for the first time, to our knowledge, extensive colonization by endophytic bacteria in innumerable numbers in the confined peri-space between the cell wall and the plasma membrane. Endophytic bacteria are normally considered to enter plants through the roots and colonize the large aerenchyma or the intercellular spaces in the roots, which form their major niche, as documented in different monocots like rice, maize, sugarcane and kallar grass (Dong *et al.* 1994; Barraquio *et al.* 1997; Reinhold-Hurek and Hurek 1998; Gyaneshwar *et al.* 2001) and in dicot systems like cotton (Hallmann *et al.* 1997), grapes (Compant *et al.* 2008, 2011) and olive (Prieto *et al.* 2011). Studies employing fluorescently labelled endophytes have shown root hairs as the primary entry point with subsequent colonization in the intercellular spaces of the root cortex (Compant *et al.* 2008; Prieto *et al.* 2011). Subsequent upward movement is facilitated through xylem reaching various plant parts and organs (Gyaneshwar *et al.* 2001; Rosenblueth and Martínez-Romero 2006; Compant *et al.* 2008, 2011). The intercellular spaces, which are often described as a habitat for bacterial endophytes, are technically covered by the term apoplast, meaning outside protoplast, as opposed to symplast, the network of cell cytoplasm interconnected by plasmodesmata (Leopold and Kriedman 1975; Sattelmacher 2001; Taiz and Zeiger 2003; Evert 2006). The region of colonization elucidated in the study has been confined to the narrow space between the cell wall and plasma membrane, which differed from the normally described apoplastic colonization composed of the intercellular spaces, xylem and vascular interconnections (Sattelmacher 2001). It is probable that the bacteria migrated from the loose intercellular spaces in the roots to the narrow intra-cell wall region in the shoots through the gaps or through the vascular interconnections. Studies on apoplast-colonizing endophytes have often adopted the collection of apoplastic fluid through techniques like centrifugation or vacuum extraction and its plating (Barraquio *et al.* 1997; Sattelmacher 2001; Asis *et al.* 2003). It is less likely that the type of intra-cell wall colonizers documented in this study could be harvested with vacuum extraction or centrifugation, as the cells get blocked by the cell wall. Studies that provide microscopic evidence of tissue colonization show them mostly in the large intercellular spaces, primarily in the roots (Barraquio *et al.* 1997; Hallmann *et al.* 1997; Sattelmacher 2001; Bacon *et al.* 2002). Unlike in the above-cited studies, we focused on the shoot-tip tissue where the host parenchymatous cells were very closely packed with no obvious intercellular spaces or air pockets. We describe this niche as ‘peri-cytoplasmic space’ or ‘peri-space’, which corresponds to periplasm, i.e. the space between the cytoplasmic and outer membranes in bacteria, particularly in the Gram-negative group (Seltmann and Holst 2002).

The observations in this study were facilitated by the use of S9 on fresh tissue sections under high magnification (×63 or ×100 objective) and live imaging where the bacterial cells could be detected easily based on their staining and/or motility. The organisms in the narrow peri-space appeared in singles rather than in groups, unlike what is documented in the studies employing TEM (Reinhold-Hurek and Hurek 1998; Gyaneshwar *et al.* 2001) or FISH (Compant *et al.* 2011) where paraffin- or resin-embedded tissue post-fixation had been used. Wherever tissue fixation is involved, there is the chance of air entrapment and free spaces if the fixation step is not conducted properly (Prieto *et al.* 2007). Lucero *et al.* (2011) employed S9–PI staining to study the endophytic microbiome in *Atriplex* sp., but adopted tissue fixation in glutaraldehyde post-staining and a low magnification (×20 objective), a resolution that was probably insufficient to detect individual bacterial cells. With the free-hand-cut sections, there was a possibility of bacterial cells becoming dislodged, but the confocal *z*-scanning over undisturbed cell layers confirmed their above-defined location inside tissue. Live imaging of plant cells has provided considerable new information about cell structure and functioning in recent years (Shaw 2006). A direct viewing of the confocal screen is highly recommended to visualize the bacterial positioning or movement in the confined peri-space as upon conversion to avi files the confocal time-lapse files gave brief-span fast video files, which was a limitation faced in presenting the real-time situation.

Propidium iodide and DAPI treatments did not normally detect the endophytic bacteria on fresh tissue sections, but did so in ethanol-challenged sets, indicating that the loss of membrane integrity was a necessity for the entry of these stains into bacterial cells (Anonymous 2004), and that healthy plant tissue contained only live bacteria. The observations from TEM supported the bacterial line-up just inside the cell wall. It is to be noted that S9–PI staining did not work effectively with the formalin- or paraformaldehyde-fixed tissue owing to high tissue auto-fluorescence or the lack of any signal. Further, the staining efficiency in phosphate buffer or PBS was low, possibly influenced by the buffer (Anonymous 2004). In addition, all the host cells in a field did not uniformly display stained bacterial cells with S9. Whether this resulted from inadequate penetration of the dye in fresh tissue or was a reflection of an uneven bacterial distribution is not clear at present. With S9 staining of pure bacterial cultures, the signal level varied with the organism, suggesting that this was possibly linked to the stage of growth or the DNA content. Altogether, the results suggested that S9 staining could be taken as a gross bacterial detection tool but not for assessing the extent of variability.

It is pertinent to note that all the suckers examined across different genotypes of banana, as well as the apparently clean micropropagated banana cultures *in vitro*, also showed bacterial presence in the cell peri-space. The wide prevalence and the sheer numbers in which the organisms are present indicate that this association could have evolved with the crop a long time ago rather than the immediate lateral recruitment. Endophytes are generally considered to be selected or recruited from the rhizosphere by the host plant (Rosenblueth and Martínez-Romero 2006; Compant *et al.* 2010; Long *et al.* 2010; Bulgarelli *et al*. 2012; Lundberg *et al*. 2012). While this may be the case for seed-propagated plants, there may be an endophytic continuum in tissue for the clonally propagated plants with the established organisms becoming an integral component through generations (Thomas and Soly 2009; Compant *et al.* 2011).

The fact that banana tissue homogenate showed abundant bacteria under direct microscopy but hardly any colony growth upon plating undertaken as part of this study (data not shown) and in the studies documented earlier (Thomas *et al.* 2008*a*; Thomas and Soly 2009) is an endorsement of the general non-culturability of the associated organisms or that their growth requirements are yet to be understood. A large proportion of endophytes associated with different plant species are considered to be not amenable for cultivation (Thomas *et al.* 2007*b*; Wang *et al.* 2008; Thomas 2011; Sessitsch *et al.* 2012). The classes or groups of organisms involved or their functions are not known at present. It is not a simple task to understand the functionality of the endophytic bacteria here considering the diversity of the organisms so far isolated from this crop in the earlier studies (Thomas *et al.* 2008*b*; Thomas and Soly 2009), the non-culturability of the large share of the organisms (Thomas *et al.* 2008*a*; Thomas and Soly 2009) and the non-feasibility of excluding the entire native microflora for studying specific organisms (Thomas 2011). While approaches like FISH will give a glimpse of bacterial diversity, it would warrant exhaustive approaches like metagenome analysis to elucidate the diversity and decipher the functions (Wang *et al.* 2008; Sessitsch *et al.* 2012). Furthermore, the use of endophytes isolated from banana after tagging with fluorescent markers such as GFP would help in studying the mode of entry and the pattern of tissue colonization, and in elucidating the functions; this is envisaged as a future study.

Although endophytic bacteria are generally considered to be extracellular colonizers except for some intra-cellular presence in the root cortex at the time of entry, intra-cellular colonization of non-root tissue has been reported in some instances. This included the colonization of shoot meristem in Scotch pine (Pirttilä *et al.* 2000) and of *in vitro* grown peach palms, the latter termed ‘bacteriosomes’ (de Almeida *et al.* 2009). Our observations employing cell suspension cultures have also indicated some intra-cellular bacterial presence, but this elucidation required a different microscopic approach than the staining of extra-cytoplasmic bacteria (P. Thomas *et al*., unpubl. res.). Although the cell wall forms an integral component in plant cells, in principle, only the cytoplasmic region constitutes the active cell machinery. Therefore, we consider the present observation of bacterial colonization as distinct from the extensively documented intercellular apoplastic colonization or the intra-cellular cytoplasmic colonization.

Detection of abundant bacteria in banana shoots assumes more importance as the shoot tissue contributes to the perpetuation of the host, including in micropropagation systems (Thomas *et al.* 2008*a*, *b*; Singh *et al.* 2011). The observations here highlight the need to take into account the vast amount of resident microflora while studying the colonization by laterally introduced organisms, which are often overlooked, such as in growth promotion and fluorescent tagging studies (Gyaneshwar *et al.* 2001; Thomas *et al.* 2007*a*; Compant *et al.* 2008; Prieto *et al.* 2011). Further, the abundant bacterial cells detected in the *in vitro* stocks of banana under a supposedly axenic tissue culture system further point to the fact that the gnotobiotic studies need to consider these non-obvious and non-culturable endophytes associated with the tissue (Thomas *et al.* 2008*a*; de Almeida *et al.* 2009; Prieto *et al.* 2011; Thomas 2011).

This study makes a significant advance in the form of elucidating the abundant bacterial colonization in the cell wall–plasma membrane peri-space with the visualization of live and active bacterial cells by adopting the Live/Dead viability staining kit, unlike in other techniques such as FISH or TEM which detect non-live bacteria. The presence of the organisms in high numbers is an indication that they play an integral role in the biology of the host, with implications for studies relating to plant physiology, molecular biology and plant microbiology. It is also of concern that the DNA from such organisms would be co-purified with plant DNA, which could significantly alter the conclusions from molecular studies (Thomas *et al.* 2007*b*, 2008*a*; Thomas and Soly 2009).

## Conclusions

This study demonstrates the applicability of the Live/Dead bacterial staining kit on non-fixed fresh tissue sections for the detection of endophytic bacteria and reveals the extensive bacterial colonization in the intra-cellular extra-cytoplasmic peri-space in banana shoot-tip tissue, which we consider as a niche not elucidated in previous studies. The microorganisms, present in innumerable numbers, possibly share a deep and integral association with the host, but show apparently mutualistic benefits with no obvious adverse or pathogenic effect on the host. The observations reported here open the path to further in-depth investigations on the plant–endophyte association and interactions.

## Sources of Funding

The study was undertaken under the AMAAS project ‘Basic and applied investigations on endophytic microorganisms in horticultural crops’ funded by the Indian Council of Agricultural Research through the National Bureau of Agriculturally Important Microorganisms (NBAIM), Mau.

## Contributions by the Authors

P.T. contributed in the development of the concept, bright-field, phase-contrast, fluorescent and confocal microscopy, and the preparation and uploading of the article. Electron microscopy work was done by K.M.R. This publication bears IIHR Contribution No. 25/2012.

## Conflict of interest statement

None declared.

## Supporting Information

The following Supporting Information is available in the online version of this article–

**Video 1.** Confocal time-lapse imaging of banana cv. Grand Naine fresh tissue section stained with SYTO 9 displaying small green-fluorescing bacteria along the periphery of cells or moving in the cell peri-space. The video was captured with the ×63 objective over 30 s and the confocal file was converted to avi format using Image J software.

**Video 2.** Confocal time-lapse imaging of banana cv. Grand Naine fresh tissue section stained with SYTO 9 displaying white fluorescing bacteria along the periphery of the cell or moving in the cell peri-space. The video was captured with the ×63 objective over 30 s and the confocal file was converted to avi format using Image J software.

**Video 3.** Confocal time-lapse imaging of banana cv. Grand Naine fresh tissue section stained with SYTO 9 displaying green-fluorescing bacteria along the periphery of the cell or moving in the cell peri-space. The video was captured with the ×63 objective over 30 s and the confocal file was converted to avi format using Image J software.

**Video 4.** Confocal *z*-stack imaging of banana cv. Grand Naine over cell layers to a depth of 40–50 µm displaying abundant bacteria stained with SYTO 9 in the cell peri-space. The video was prepared with the help of Windows Movie Maker, assembling the *z*-stacks from different planes generated with the use of LSM image browser (×63 objective).

**Video 5.** Confocal *z*-stack imaging of banana cv. Grand Naine over cell layers displaying abundant fluorescing bacteria after staining with SYTO 9. The video was captured with the ×63 objective to a depth of 50 µm and the confocal file was converted to avi format using Image J software.

**Video 6.** Confocal *z*-stack imaging of banana cv. Robusta over several cell layers, displaying abundant bacteria stained with SYTO 9 confined to the cell peri-space. The video was prepared with the help of Windows Movie Maker by assembling the *z*-stacks from different planes generated with the use of LSM image browser (×63 objective).

**Figure S1.** Grand Naine tissue sections under bright-field and phase-contrast microscopy. Aseptically prepared thin (∼50–100 µm) free-hand-cut tissue sections from the shoot-tip explants of banana cv. Grand Naine displaying intact host cells with internal organelles including plastids and mitochondria with no obvious intercellular spaces under bright-field (A) and phase-contrast microscopy (B) under ×100 objective (horizontal bar = 2 µm).

**Figure S2.** Effect of tissue fixation or the sample mounting in phosphate buffer during epifluorescence microscopy with SYTO 9 stock prepared in water. Control tissue with no auto-fluorescence (A), fresh tissue sections mounted in water with S9 (B), tissue section from formalin-fixed tissue (C), or paraformaldehyde-fixed tissue (D), tissue section mounted in 0.5 M PO_4_ buffer (E) or in PBS (F). Images captured with the 100× objective of the epifluorescence microscope with 2 s exposure for control tissue and <50 ms for the other samples (horizontal bar = 5 µm).

**Figure S3.** Confocal *z*-stacking after SYTO 9 staining of fresh tissue sections of cv. Grand Naine. Aseptically prepared, thin (∼50–100 µm) free-hand-cut tissue sections from the shoot-tip explants of banana cv. Grand Naine stained with SYTO 9 displaying abundant bacteria along the cell periphery over different cell layers in confocal laser scanning microscopy. (A), (B), (C), (D), (E) and (F) correspond to *z*-stacks at 1, 7, 12, 17, 19 and 24 µm, respectively, from the edge of the sampled tissue (horizontal bar = 5 µm).

**Figure S4.** Confocal images of pure cultures of different bacteria. Endophytic bacterial isolates from banana after SYTO 9 staining: coccus-shaped *Brachybacterium*, *Micrococcus luteus*, *Kocuria rosea*, *Staphylococcus epidermidis* (A–D); fine rod-shaped *Brevibacterium*, *Microbacterium* and *Tetrasphera* spp. (E–G); medium-long rods of *Enterobacter cloacae* (H); and long-rod-shaped *Bacillus subtilis* (I) gathered from 2-day-old trypticasein soy agar plate cultures at 30 °C (horizontal bar = 5 µm).

**Figure S5.** Confocal *z*-stacks of cv. Ney Poovan tissue sections after SYTO 9 staining. The image shows abundant bacteria along the cell periphery in different cell layers over 63 µm at 2-µm intervals (horizontal bar = 10 µm).

Additional Information

## References

[PLT011C1] Anonymous (2004). LIVE/DEAD® BacLight. Bacterial viability kits, product information MP 07007..

[PLT011C2] Asis CA, Adachi K, Sugimoto A, Ujihara K, Terajima Y, Fukuhara S (2003). Population of diazotrophic endophytes in stem apoplast solution of sugarcane and related grass species in Tanegashima, Japan. Microbes and Environments.

[PLT011C3] Backman PA, Sikora RA (2008). Endophytes: an emerging tool for biological control. Biological Control.

[PLT011C4] Bacon CW, Glenn AE, Hinton DM, Hurst CJ, Crawford RL, McInerney MJ, Knudsen GR, Stetzenbach LD (2002). Isolation, in planta detection and culture of endophytic bacteria and fungi. Manual of environmental microbiology.

[PLT011C5] Barraquio WL, Revilla L, Ladha JK (1997). Isolation of endophytic diazotrophic bacteria from wetland rice. Plant and Soil.

[PLT011C6] Berney M, Hammes F, Bosshard F, Weilenmann H-U, Egli T (2007). Assessment and interpretation of bacterial viability by using the Live/Dead BacLight kit in combination with flow cytometry. Applied and Environmental Microbiology.

[PLT011C7] Böhm M, Hurek T, Reinhold-Hurek B (2007). Twitching motility is essential for endophytic rice colonization by the N_2_-fixing endophyte *Azoarcus* sp. strain BH72. Molecular Plant-Microbe Interactions.

[PLT011C45] Bulgarelli D, Rott M, Schlaeppi K, van Themaat EVL, Ahmadinejad N, Assenza F, Rauf P, Huettel B, Reinhardt R, Schmelzer E, Peplies J, Gloeckner FO, Amann R, Eickhorst T, Schulze-Lefert P (2012). Revealing structure and assembly cues for *Arabidopsis* root-inhabiting bacterial microbiota. Nature.

[PLT011C9] Compant S, Kaplan H, Sessitsch A, Nowak J, Ait Barka E, Clément C (2008). Endophytic colonization of *Vitis vinifera* L. by *Burkholderia phytofirmans* strain PsJN: from the rhizosphere to inflorescence tissues. FEMS Microbiology Ecology.

[PLT011C8] Compant S, Clément C, Sessitsch A (2010). Plant growth-promoting bacteria in the rhizo- and endosphere of plants: their role, colonization, mechanisms involved and prospects for utilization. Soil Biology and Biochemistry.

[PLT011C10] Compant S, Mitter B, Colli-Mull JG, Gangl H, Sessitsch A (2011). Endophytes of grapevine flowers, berries and seeds: identification of cultivable bacteria, comparison with other plant parts, and visualization of niches of colonization. Microbial Ecology.

[PLT011C11] de Almeida CV, Andreote FD, Yara R, Tanaka FAO, Azevedo JL, de Almeida M (2009). Bacteriosomes in axenic plants: endophytes as stable endosymbionts. World Journal of Microbiology and Biotechnology.

[PLT011C12] Dong Z, Canny MJ, McCully ME, Roboredo MR, Cabadilla CF, Ortega E, Rodes R (1994). A nitrogen-fixing endophyte of sugarcane stems: a new role for the apoplast. Plant Physiology.

[PLT011C13] Doty SL (2008). Enhancing phytoremediation through the use of transgenics and endophytes. New Phytologist.

[PLT011C14] Evert RF (2006). Esau's plant anatomy.

[PLT011C15] Gião MS, Wilks SA, Azevedo NF, Vieira MJ, Keevil CW (2009). Validation of SYTO 9/propidium iodide uptake for rapid detection of viable but noncultivable *Legionella pneumophila*. Microbial Ecology.

[PLT011C16] Gyaneshwar P, James EK, Mathan N, Reddy PM, Reinhold-Hurek B, Ladha JK (2001). Endophytic colonization of rice by a diazotrophic strain of *Serratia marcescens*. Journal of Bacteriology.

[PLT011C17] Hallmann J, Quadt-Hallmann A, Mahaffe WF, Kloepper JW (1997). Bacterial endophytes in agricultural crops. Canadian Journal of Microbiology.

[PLT011C18] Hardoim PR, van Overbeek LS, van Elsas JD (2008). Properties of bacterial endophytes and their proposed role in plant growth. Trends in Microbiology.

[PLT011C19] Kaewkla O, Franco CMM (2013). Rational approaches to improving the isolation of endophytic actinobacteria from Australian native trees. Microbial Ecology.

[PLT011C20] Leopold AC, Kriedman PE (1975). Plant growth and development.

[PLT011C21] Long HH, Sonntag DG, Schmidt DD, Baldwin IT (2010). The structure of the culturable root bacterial endophyte community of *Nicotiana attenuata* is organized by soil composition and host plant ethylene production and perception. New Phytologist.

[PLT011C22] Lucero ME, Unc A, Cooke P, Dowd S, Sun S (2011). Endophytic microbiome diversity in micropropagated *Atriplex canescens* and *Atriplex torreyi* var *giffithsii*. PLoS One.

[PLT011C46] Lundberg DS, Lebeis SL, Paredes SH, Yourston S, Gehring J, Malfatti S, Tremblay J, Engelbrektson A, Kunin V, del Rio TG, Edgar RC, Eickhorst T, Ley RE, Hugenholtz P, Tringe SG, Dangl JL (2012). Defining the core *Arabidopsis thaliana* root microbiome. Nature.

[PLT011C23] Mano H, Morisaki H (2008). Endophytic bacteria in the rice plant. Microbes and Environments.

[PLT011C24] Parniske M (2000). Intracellular accommodation of microbes by plants: a common developmental program for symbiosis and disease?. Current Opinion in Plant Biology.

[PLT011C25] Pirttilä AM, Laukkanen H, Pospiech H, Myllyä R, Hohtola A (2000). Detection of intracellular bacteria in the buds of Scotch pine (*Pinus sylvestris* L.) by *in situ* hybridization. Applied and Environmental Microbiology.

[PLT011C26] Prieto P, Moore G, Shaw P (2007). Fluorescence *in situ* hybridization on vibratome sections of plant tissues. Nature Protocols.

[PLT011C27] Prieto P, Schilirò E, Maldonado-González MM, Valderrama R, Barroso-Albarracín JB, Mercado-Blanco J (2011). Root hairs play a key role in the endophytic colonization of olive roots by *Pseudomonas* spp. with biocontrol activity. Microbial Ecology.

[PLT011C28] Reinhold-Hurek B, Hurek T (1998). Life in grasses: diazotrophic endophytes. Trends in Microbiology.

[PLT011C29] Reinhold-Hurek B, Hurek T (2011). Living inside plants: bacterial endophytes. Current Opinion in Plant Biology.

[PLT011C30] Rosenblueth M, Martínez-Romero E (2006). Bacterial endophytes and their interactions with hosts. Molecular Plant-Microbe Interactions.

[PLT011C31] Ryan R, Germaine K, Franks A, Ryan DJ, Dowling DN (2008). Bacterial endophytes: recent development and applications. FEMS Microbiology Letters.

[PLT011C32] Sattelmacher B (2001). The apoplast and its significance for plant mineral nutrition. New Phytologist.

[PLT011C33] Seltmann G, Holst O (2002). The bacterial cell wall.

[PLT011C34] Sessitsch A, Hardoim P, Döring J, Weilharter A, Krause A, Woyke T, Mitter B, Hauberg-Lotte L, Friedrich F, Rahalkar M, Hurek T, Sarkar A, Bodrossy L, van Overbeek L, Brar D, van Elsas JD, Reinhold-Hurek B (2012). Functional characteristics of an endophyte community colonizing rice roots as revealed by metagenomic analysis. Molecular Plant-Microbe Interactions.

[PLT011C35] Shaw SL (2006). Imaging the live plant cell. The Plant Journal.

[PLT011C36] Singh HP, Uma S, Selvarajan R, Karihaloo JL (2011). Micropropagation for production of quality banana planting material in Asia Pacific.

[PLT011C37] Taiz L, Zeiger E (2003). Plant physiology.

[PLT011C38] Thomas P (2011). Intense association of non-culturable endophytic bacteria with antibiotic-cleansed in vitro watermelon and their activation in degenerating cultures. Plant Cell Reports.

[PLT011C39] Thomas P, Soly TA (2009). Endophytic bacteria associated with growing shoot tips of banana (*Musa* sp.) cv. Grand Naine and the affinity of endophytes to the host. Microbial Ecology.

[PLT011C40] Thomas P, Kumari S, Swarna GK, Gowda TKS (2007a). Papaya shoot tip associated endophytic bacteria isolated from *in vitro* cultures and host–endophyte interaction in vitro and in vivo. Canadian Journal of Microbiology.

[PLT011C41] Thomas P, Kumari S, Swarna GK, Prakash DP, Dinesh MR (2007b). Ubiquitous presence of fastidious endophytic bacteria in field shoots and index-negative apparently clean shoot-tip cultures of papaya. Plant Cell Reports.

[PLT011C42] Thomas P, Swarna GK, Patil P, Rawal RD (2008a). Ubiquitous presence of normally non-cultivable endophytic bacteria in field shoot-tips of banana and their gradual activation to quiescent cultivable form in tissue cultures. Plant Cell, Tissue and Organ Culture.

[PLT011C43] Thomas P, Swarna GK, Roy PK, Patil P (2008b). Identification of cultivable and originally non-culturable endophytic bacteria isolated from shoot tip cultures of banana cv. Grand Naine. Plant Cell, Tissue and Organ Culture.

[PLT011C44] Wang H-X, Geng Z-L, Zeng Y, Shen Y-M (2008). Enriching plant microbiota for a metagenomic library construction. Environmental Microbiology.

