# Supplementary material for: Microscopic elucidation of abundant endophytic bacteria colonizing the cell wall–plasma membrane peri-space in the shoot-tip tissue of banana
Source: AoB Plants. 2013 Feb 22;5:plt011. doi: 10.1093/aobpla/plt011 (PMC4455319; doi:10.1093/aobpla/plt011)
Supplement: Additional Information [file supp_5_plt011_index.html]

Additional Information 

# Microscopic elucidation of abundant endophytic bacteria colonizing the cell wall–plasma membrane peri-space in the shoot-tip tissue of banana

## Additional Information

**Files in this Data Supplement:**

- Additional Information - doc file
- Additional Information - mp4 file (Video 1)
- Additional Information - mp4 file (Video 2)
- Additional Information - mp4 file (Video 3)
- Additional Information - mp4 file (Video 4)
- Additional Information - mp4 file (Video 5)
- Additional Information - mp4 file (Video 6)
- Additional Information - jpg file (Figure S1)
- Additional Information - jpg file (Figure S2)
- Additional Information - jpg file (Figure S3)
- Additional Information - jpg file (Figure S4)
- Additional Information - jpg file (Figure S5)
