# Supplementary material for: Microscopic elucidation of abundant endophytic bacteria colonizing the cell wall–plasma membrane peri-space in the shoot-tip tissue of banana
Source: AoB Plants. 2013 Feb 22;5:plt011. doi: 10.1093/aobpla/plt011 (PMC4455319; doi:10.1093/aobpla/plt011)
Supplement: Additional Information [file supp_plt011_pls011supp.doc]

**SUPPORTING INFORMATION**

The following **[SUPPORTING INFORMATION**] is available in the on-line version of this article

**Video 1.** Confocal time-lapse imaging of banana cv. Grand Naine fresh tissue section applied with SYTO 9 displaying small green-fluorescing bacteria along the periphery of cells or moving in the cell peri-space. The video was captured with the 63× objective over 30 s and the confocal file was converted to avi format using Image J software.

**Video 2.** Confocal time-lapse imaging of banana cv. Grand Naine fresh tissue section applied with SYTO 9 displaying white fluorescing bacteria along the periphery of the cell or moving in the cell peri-space. The video was captured with the 63× objective over 30 s and the confocal file was converted to avi format using Image J software.

**Video 3**. Confocal time-lapse imaging of banana cv. Grand Naine fresh tissue section applied with SYTO 9 displaying green fluorescing bacteria along the periphery of the cell or moving in the cell peri-space. The video was captured with the 63× objective over 30 s and the confocal file was converted to avi format using Image J software.

**Video 4.** Confocal z-stack imaging of banana cv. Grand Naine over cell layers to a depth of 40-50 µm indicating abundant bacteria stained with SYTO 9 in the cell peri-sapce. The video was prepared with the help of windows movie maker assembling the z-stacks from different planes generated with the use of LSM image browser (63× objective).

**Video 5.** Confocal z-stack imaging of banana cv. Grand Naine over cell layers displaying abundant fluorescing bacteria after staining with SYTO 9. The video was captured with the 63× objective to a depth of 50 µm and the confocal file was converted to avi format using Image J software.

**Video 6.** Confocal z-stack imaging of banana cv. Robusta over several cell layers indicating abundant bacteria stained with SYTO 9 confined to the cell peri-space. The video was prepared with the help of windows movie maker by assembling the z-stacks from different planes generated with the use of LSM image browser (63× objective).

**Figure S1. Grand Naine tissue sections under bright field and phase contrast microscopy.** Aseptically prepared thin (approx. 50-100 µm) free hand tissue sections from the shoot-tip explants of banana cv. Grand Naine displaying intact host cells with internal organelles including plastids and mitochondria with no obvious intercellular spaces under bright field (A) and phase contrast microscopy (B) under 100× objective (horizontal bar = 2 µm)

**Figure S2.** **Effect of tissue fixation or the sample mounting in phosphate buffer during epifluorescence microscopy with SYTO 9 stock prepared in water.**  Control tissue with no auto-fluorescence (A), fresh tissue sections mounted in water with S9 (B), tissue section from formalin fixed tissue (C), or paraformaldehyde fixed-tissue (D), tissue section mounted in 0.5 M PO_4_ buffer (E) or in PBS (F). Images captured with the 100× objective of epi fluorescence microscope with 2 s exposure for control tissue and < 50 milli seconds for the other samples (horizontal bar = 5 µm).

**Figure S3. Confocal z-stacking after SYTO 9 staining of fresh tissue sections of cv. Grand Naine.** Aseptically prepared thin (approx. 50-100 µm) free hand tissue sections from the shoot-tip explants of banana cv. Grand Naine stained with SYTO 9 displaying abundant bacteria along the cell periphery over different cell layers in confocal laser scanning microscopy. Panels A, B, C, D, E and F correspond to z-stacks at 1, 7, 12, 17, 19 and 24 µm from the edge of sampled tissue (horizontal bar = 5 µm).

**Figure S4. Confocal images of pure cultures of different bacteria.** Endophytic bacterial isolates from banana after SYTO 9 staining; cocci shaped *Brachybacterium*, *Micrococcus luteus,* *Kocuria rosea*, *Staphylococcus* *epidermidis* (A-D); fine rod shaped *Brevibacterium*, *Microbacterium* and *Tetrasphera* spp. (E-G), medium-long rods of *Enterobacter cloacae* (H) and long-rod shaped *Bacillus subtilis* (I) gathered from 2 day-old trypticasein soy agar plate cultures at 30°C (horizontal bar = 5 µm).

**Figure S5. Confocal z-stacks of cv. Ney Poovan tissue sections after SYTO 9 staining.** The image shows abundant bacteria along the cell periphery in different cell layers over 63 µm at 2 µm intervals (horizontal bar = 10 µm).
