# Supplementary figures and images for: Microscopic elucidation of abundant endophytic bacteria colonizing the cell wall–plasma membrane peri-space in the shoot-tip tissue of banana
Source: AoB Plants. 2013 Feb 22;5:plt011. doi: 10.1093/aobpla/plt011 (PMC4455319; doi:10.1093/aobpla/plt011)

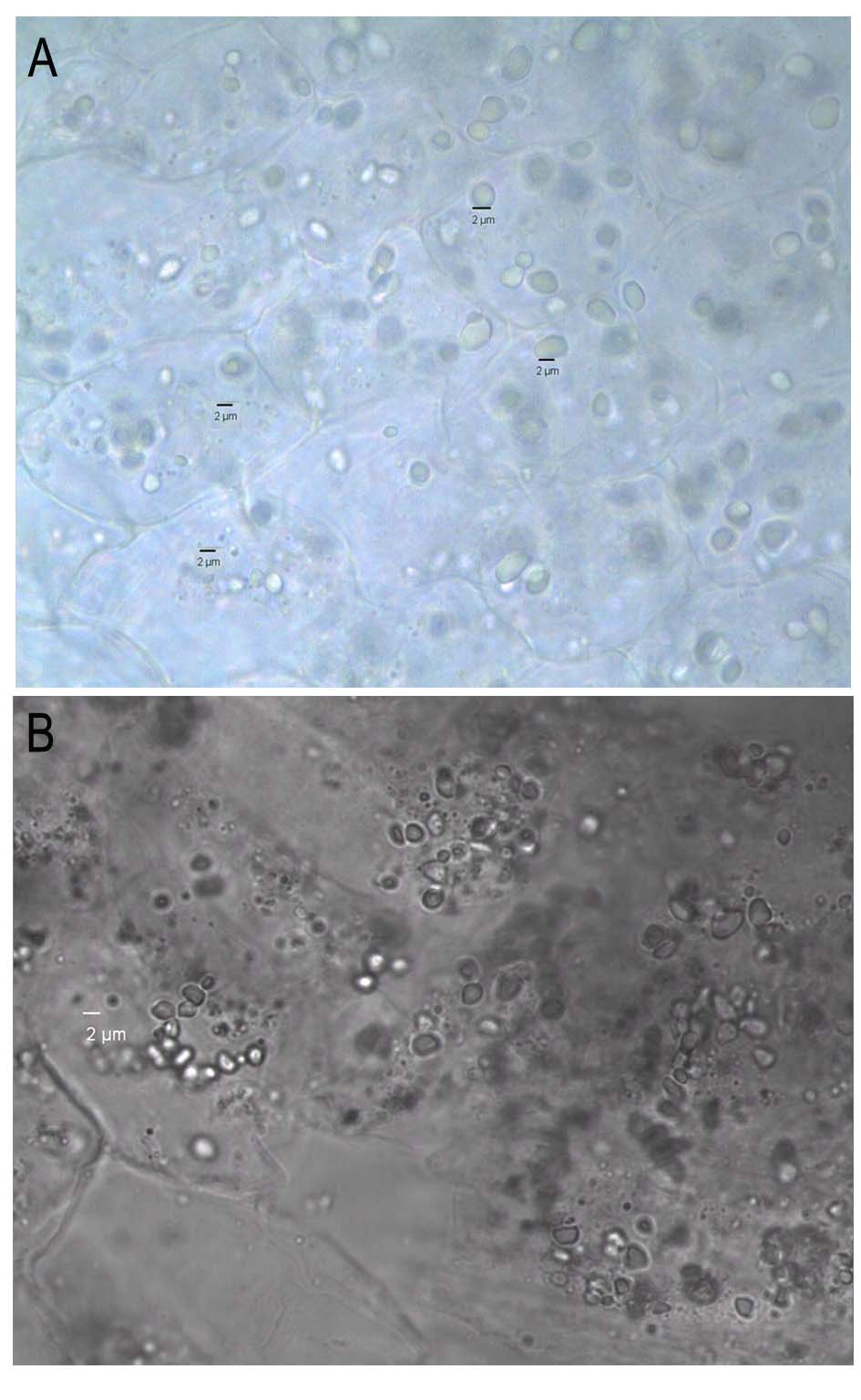

Supplement: Additional Information [file supp_plt011_plt011supp_fig1.jpg]

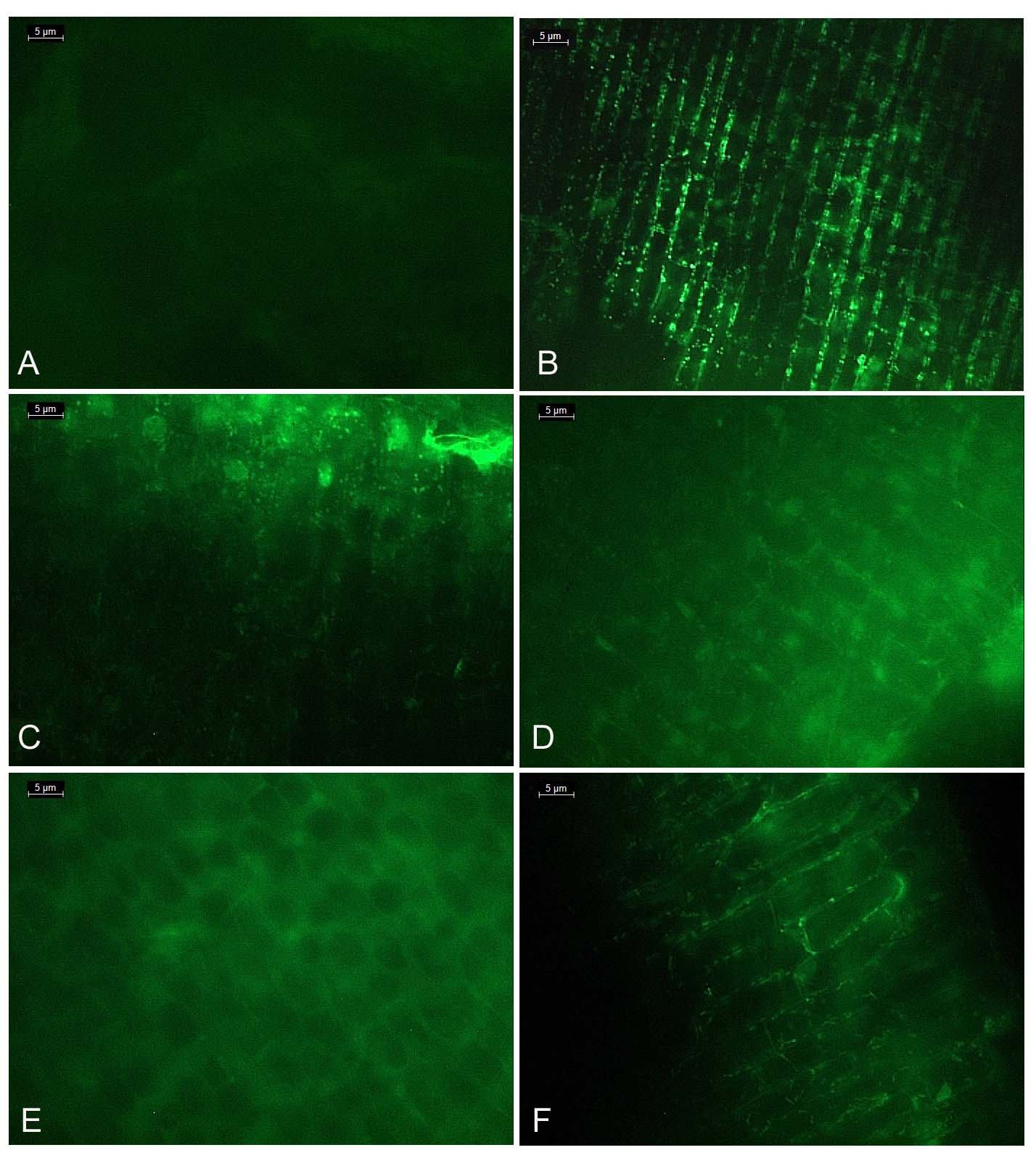

Supplement: Additional Information [file supp_plt011_plt011supp_fig2.jpg]

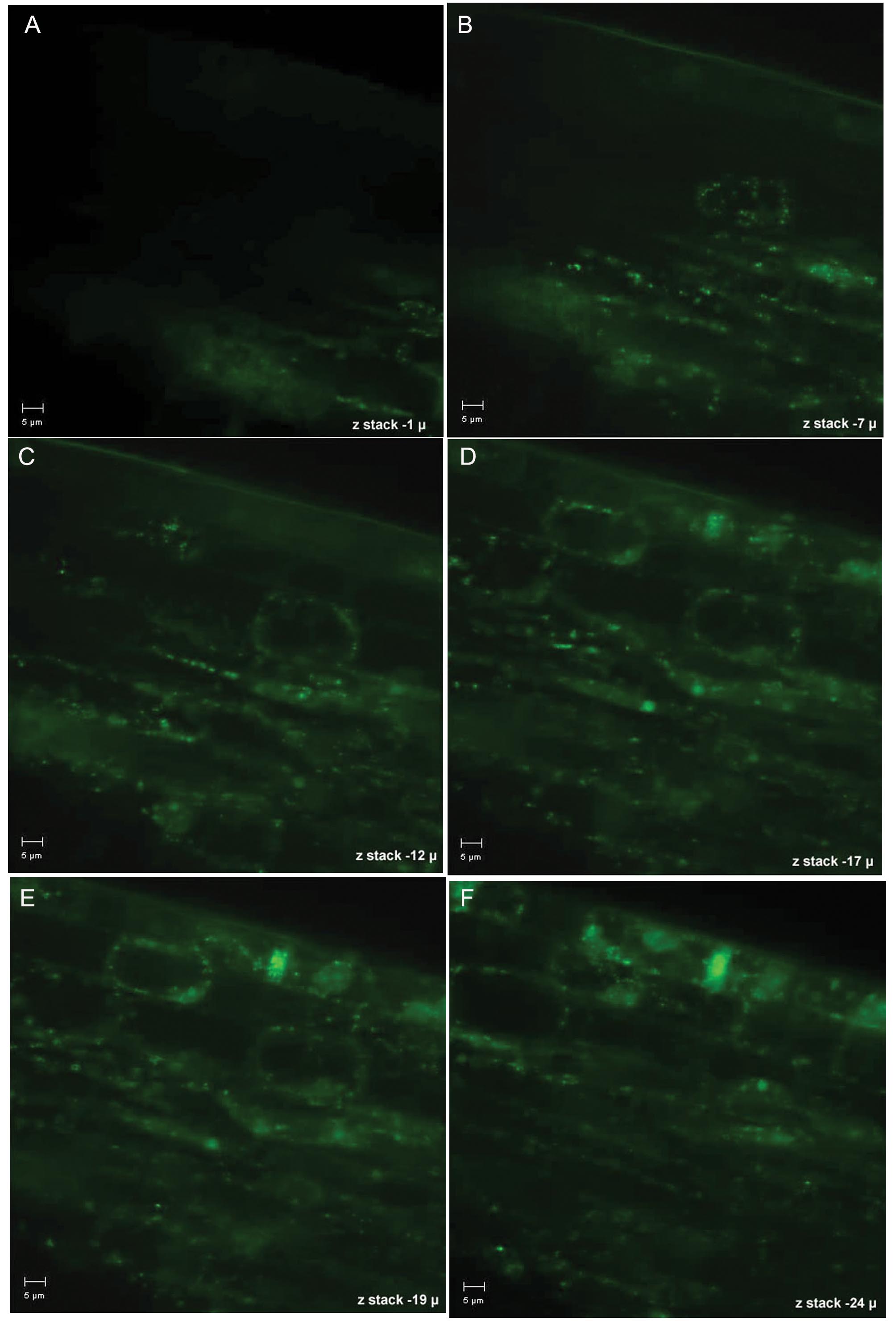

Supplement: Additional Information [file supp_plt011_plt011supp_fig3.jpg]

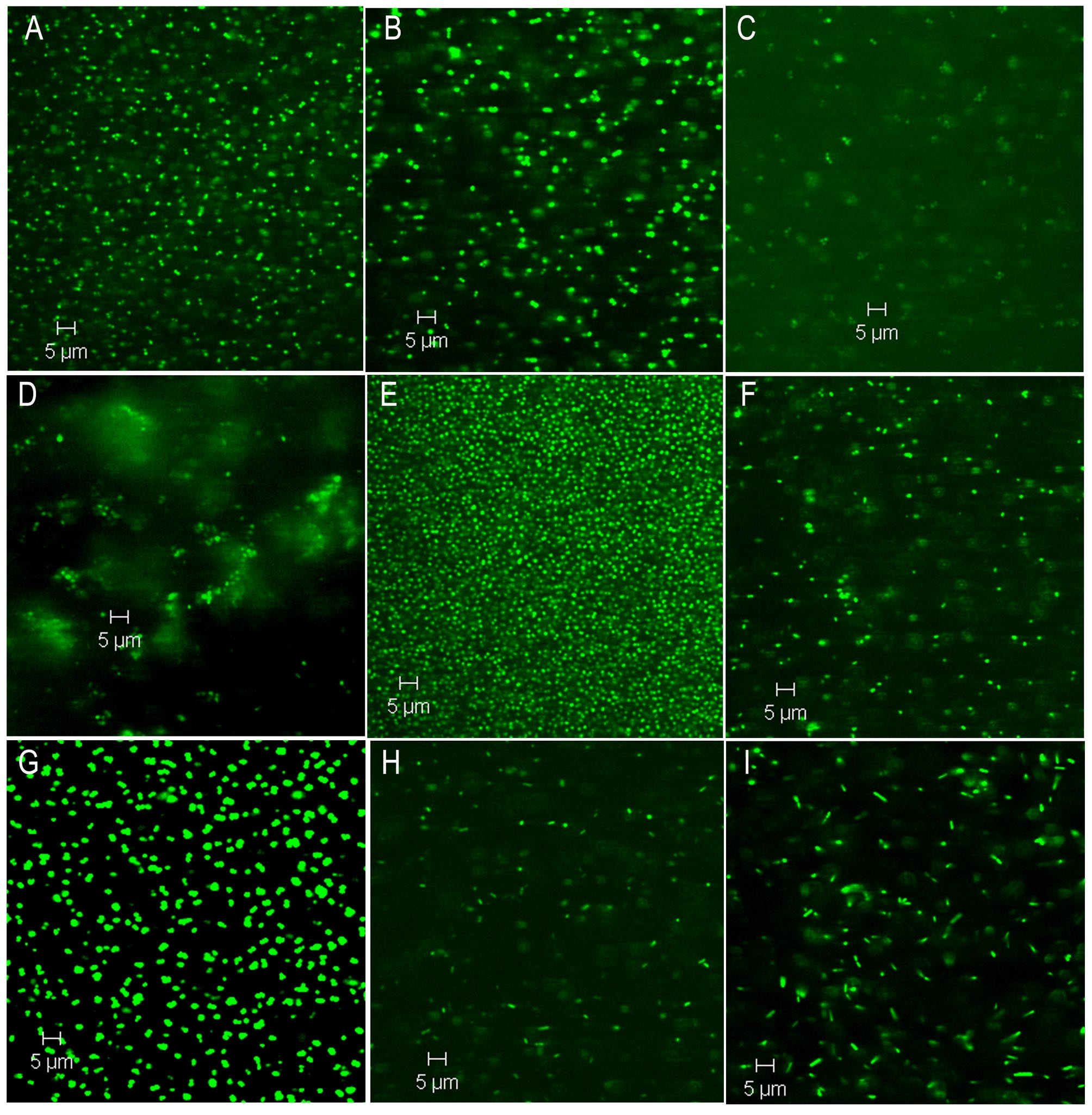

Supplement: Additional Information [file supp_plt011_plt011supp_fig4.jpg]

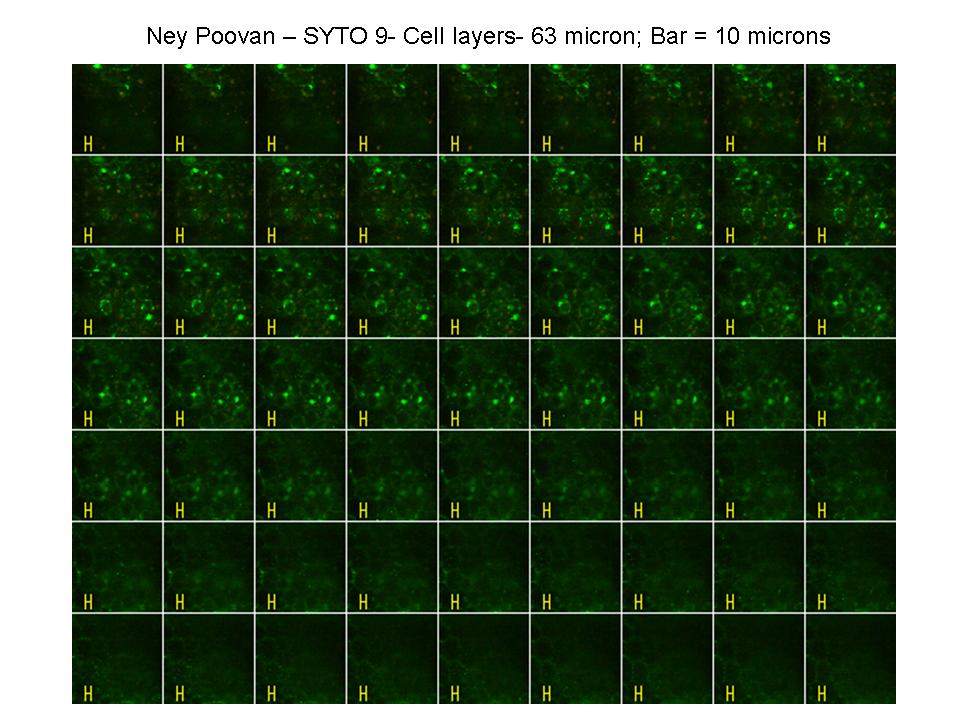

Supplement: Additional Information [file supp_plt011_plt011supp_fig5.jpg]
